# Association Between Vertebral Bone Quality Score and Dual-Energy X-ray Absorptiometry for the Assessment of Bone Mineral Density in Adolescent Patients

**DOI:** 10.7759/cureus.53402

**Published:** 2024-02-01

**Authors:** Meghna Patel, Jacob Razzouk, David Shin, Andrew J Cabrera, Kai Nguyen, Alex Bouterse, Paddington Mbumbgwa, Zachary Brandt, Wayne Cheng, Olumide Danisa, Omar Ramos

**Affiliations:** 1 School of Medicine, University of California, Riverside, Riverside, USA; 2 School of Medicine, Loma Linda University, Loma Linda, USA; 3 Department of Orthopedic Surgery, Jerry L. Pettis Veterans Affairs (VA) Medical Center, Loma Linda, USA; 4 Department of Orthopedic Surgery, Loma Linda University, Loma Linda, USA; 5 Department of Spine Surgery, Twin Cities Spine Center, Minneapolis, USA

**Keywords:** dual-energy x-ray absorptiometry, dexa, adolescent, vbq, vertebral bone quality

## Abstract

Background: The MRI-based vertebral bone quality (VBQ) score is an assessment tool for bone mineral density (BMD) that has been validated in adults against the clinical standard of dual-energy X-ray absorptiometry (DEXA). However, VBQ has yet to be validated against DEXA for use in adolescents. This study evaluated the associations between adolescent VBQ scores, DEXA Z-scores, and BMD values.

Methods: The radiographic records of 63 consecutive patients between the ages of 11 and 21 who underwent MRI of the abdomen and pelvis and DEXA of the spine and hip were retrieved. The collected radiographic data consisted of the MRI-based VBQ score, DEXA Z-score, and BMD values of the femoral neck, L1-4 vertebrae, and total body. The VBQ score was calculated by taking the median signal intensity (MSI) from L1-L4 and the SI of the L3 cerebrospinal fluid (CSF). The VBQ score was derived as the quotient of MSI_L1-L4_ divided by SI_CSF_.

Results: A mean VBQ score of 2.41 ± 0.29 was observed. Strong correlations of -0.749 (p<0.0001) and -0.780 (p<0.0001) were detected between the VBQ score and DEXA femoral neck and spine Z-scores, respectively. Correlations between VBQ score and DEXA femoral neck, spine, and total body BMD scores were -0.559 (p<0.0001), -0.611 (p<0.0001), and -0.516 (p<.0001), respectively. No significant correlations were found between the VBQ score and age, BMI, weight, or height. A mean difference in VBQ score of -0.155 (p=0.035) was observed between sexes. VBQ demonstrated moderate predictive ability for DEXA-derived Z-scores and BMD scores.

Conclusions: VBQ scores were strongly correlated with DEXA Z-scores and moderately correlated with BMD values. The VBQ score can also be used by adolescent patients as an accessory tool to assess bone health.

## Introduction

Bone mass acquisition during adolescence can determine ultimate bone health in adulthood. Peak bone mass, likely reached after late adolescence, is a significant predictor of developing osteoporosis in adulthood [1]. Therefore, factors impacting bone mass acquisition during childhood or adolescence can contribute to future risk for osteoporosis and fragility fracture.

Bone mineral density (BMD), derived from dual-energy X-ray absorptiometry (DEXA), is the clinical standard for the diagnosis of osteoporosis and the assessment of fracture risk [2,3]. The World Health Organization defines bone density levels using a T-score that compares the patient's BMD to the average BMD of a young adult (healthy Caucasian females aged 20-29 years) [4]. A normal BMD is within one standard deviation (SD) of the young adult mean. Osteopenia is between -1.0 and -2.5 SD from the mean, while osteoporosis is defined as a T-score of -2.5 or lower than the SD. Severe osteoporosis is defined as a T-score less than -2.5 SD below the mean in conjunction with one or more osteoporotic fractures. Though widely used, DEXA has limitations in predicting fracture risk. Shuiet et al. found overlapping DEXA-derived BMD scores in individuals with and without osteoporosis who demonstrated non-vertebral fractures [5]. This highlights the need for a more sensitive risk assessment tool for fragility fractures.

Other imaging techniques, such as quantitative computed tomography (QCT) and magnetic resonance imaging (MRI), have been investigated to evaluate BMD and fracture risk [6]. Though QCT has a moderately high radiation dose, it has been shown to more precisely track bone size and shape changes that occur during adolescence [7]. Additionally, several studies have demonstrated the utility of computed tomography (CT) as an opportunistic screening tool for osteoporosis and fracture risk [8-10]. On the other hand, Ehresman et al. described the use of MRI for adult BMD assessment [11]; they were the first to create a simple MRI-based score, the vertebral bone quality (VBQ) score, that was found to be a significant predictor of healthy versus osteopenic/osteoporotic bone. The VBQ score, a validated MRI-based tool, is based on evidence demonstrating a higher T1 signal intensity in patients with osteoporosis due to decreased amounts of trabecular bone and increased intravertebral fat fraction [12,13]. The VBQ score has been shown to correlate with DEXA-derived BMD values [14], predict fragility fractures independent of DEXA-derived BMD [15], and predict vertebral compression fractures in those with spinal metastases [11]. In 2022, Ramos et al. were the first to evaluate BMD in pediatric patients with adolescent idiopathic scoliosis (AIS) using VBQ and found that those with AIS had a significantly higher VBQ score and bone density compared to match controls [16].

The VBQ score, to the best of our knowledge, has yet to be correlated against the DEXA score in adolescent patients. The goal of this current study is to evaluate the association between the MRI-based VBQ score, DEXA-derived Z-score, and BMD values in adolescent patients.

## Materials and methods

Following Loma Linda University Health Institutional Review Board approval (approval number: 5220185) in 2022, we retrieved the medical and radiographic records of 63 patients who underwent MRI of the abdomen and pelvis and DEXA of the spine and hip within one year of each other between January 2013 and June 2022. Patient consent was not required due to the retrospective nature of the study design. All patients meeting the inclusion criteria for the study were adolescents between 11 and 21 years of age and had completed an MRI and DEXA scan within one year of each other. Exclusion criteria included patients with poor-quality MRI due to motion artifacts or those with previous spinal instrumentation. The collected demographic and anthropometric data from the electronic medical record consisted of age, sex, race, height, weight, and BMI. The IMPAX6 picture archiving and communication system was utilized for all radiographic data collection (Agfa-Gavaert, Mortsel, Belgium).

Collected radiographic data included the MRI-based VBQ score, DEXA-derived Z-scores, and BMD scores of the femoral neck, trochanter (troch), intertrochanteric region (inter), total hip, total body, and L1-4 vertebrae. VBQ was measured as described by Ehresman et al. using non-contrast, T1-weighted MRI with mid-sagittal T2-weighted MRI sequences utilized as visual aids for identification of the cerebrospinal fluid (CSF). The VBQ score was calculated as described by Ehresman et al. by first placing regions of interest within the medullary portions of the L1-L4 vertebral bodies and the CSF space at the level of L3 [11]. The median signal intensity (MSI) from L1-L4 was then calculated, and the VBQ score was derived as the quotient of MSI_L1-L4_ divided by SI_CSF_. In other words, VBQ = (MSI_L1-L4_ /SI_CSF_). This metric using SI_CSF_ at L3 allows the metric to be generalizable across multiple MR systems from distinct manufacturers while still correlating with BMD as measured by DEXA scores [15].

Statistical analysis

Data collection and visualization were performed using Microsoft Excel version 16.58 (Microsoft Corporation, 2022, Redmond, WA, USA). SPSS Statistics version 28 (IBM Corp. Released 2021. IBM SPSS Statistics for Windows, Version 28.0. Armonk, NY: IBM Corp) was utilized for all subsequent statistical analyses. Q-Q plots and Shapiro-Wilk and Kolmogorov-Smirnov tests were used to assess the normality of the data. Descriptive statistics utilized means and standard deviations (SD), mean differences (MD) and standard error differences (SED), 95% confidence intervals (CI), and percentages for demographic and anthropometric data. Independent sample t-tests were used to identify sex differences for radiographic variables. Pearson’s correlation tests were constructed to identify associations among all permutations of radiographic, demographic, and anthropometric variables. Enter-method univariate linear regression models with one-way analysis of variance (ANOVA) were constructed to evaluate the predictive capacity of VBQ with respect to DEXA-derived values. Statistical significance was defined as p<0.05 for all analyses apart from regression, where alpha was set to 0.20. Homoscedasticity was assessed using homogeneity of variance tests and regression residual plots. Pearson correlation coefficients were categorized as weak, moderate, and strong, corresponding to value ranges of 0-0.3, 0.3-0.7, and 0.7-1, respectively.

## Results

We evaluated 30 female and 33 male subjects in our study. The mean age was 16.94 years, and the mean BMI was 24.6 kg/m^2^. Detailed demographic and anthropometric characteristics are displayed in Table 1. A mean VBQ score of 2.41 ± 0.29 was observed (Table 2). Significant correlations were observed between VBQ and DEXA values, though no correlations were found between VBQ scores and age, BMI, weight, or height (Table 3). A VBQ score MD of -0.155 (p=0.035) was observed based on sex (Table 4), such that female patients had a higher VBQ score than male patients. No statistically significant differences were found between Caucasian and Hispanic subjects with respect to VBQ or DEXA values. VBQ was observed to demonstrate moderate predictive ability for DEXA-derived Z-scores (Table 5) and BMD scores (Table 6). Though the VBQ score has not been validated for use in the adolescent population, we are unable to draw any strong conclusions; however, given the strong negative correlation seen between the VBQ score and the DEXA-derived Z-scores, it appears that this method shows promise in its ability to measure bone density in adolescents.

**Table 1:** Patient characteristics and reason for DEXA screening (n=63) BMI: body mass index; DEXA: dual-energy X-ray absorptiometry; SD: standard deviation

Characteristic	Mean ± SD or percentage
Age	16.94 (3.53)
BMI	24.58 (10.27)
Height (cm)	160.28 (20.57)
Weight (kg)	61.08 (19.18)
Female (%)	47.6%
Race and ethnicity	
Caucasian	30.2%
Hispanic/Latino	57.1%
Black/African American	6.3%
Asian	1.6%
Other	4.8%
Reason for DEXA	Percentage (n)
History of fracture(s)	61.9% (39)
Crohn’s disease	12.7% (8)
Ulcerative colitis	9.5% (6)
Chronic kidney disease	4.8% (3)
Lipodystrophy	4.8% (3)
History of bone marrow transplant	3.1% (2)
Polyostotic fibrous dysplasia	1.6% (1)
Niemann pick disease	1.6% (1)

**Table 2:** Mean VBQ and DEXA values BMD: body mineral density; SD: standard deviation; VBQ: vertebral bone quality; troch: trochanter; inter: intertrochanteric region; DEXA: dual-energy X-ray absorptiometry

Score	Total (n=63)		Male (n=33)		Female (n=30)	
	Mean	± SD	Mean	± SD	Mean	± SD
VBQ score	2.41	0.29	2.34	0.32	2.49	0.23
Z-score: femoral neck	-1.31	1.69	-1.17	1.81	-1.46	1.56
Z-score: troch	-1.39	1.71	-0.96	1.96	-1.87	1.26
Z-score: inter	-1.13	1.75	-0.68	1.99	-1.63	1.30
Z-score: total hip	-1.10	1.78	-0.62	2.02	-1.64	1.31
Z-score: L1	-1.11	1.46	-0.91	1.48	-1.32	1.44
Z-score: L2	-1.06	1.45	-0.81	1.56	-1.33	1.28
Z-score: L3	-0.89	1.41	-0.72	1.44	-1.07	1.38
Z-score: L4	-1.12	1.41	-0.82	1.47	-1.46	1.30
Z-score: L1-L4 total	-0.85	1.48	-0.64	1.49	-1.08	1.46
BMD: femoral neck	0.71	0.27	0.73	0.28	0.68	0.26
BMD: troch	0.67	0.21	0.69	0.22	0.65	0.21
BMD: inter	0.54	0.21	0.56	0.21	0.52	0.22
BMD: total hip	0.58	0.28	0.63	0.32	0.52	0.22
BMD: L1	0.81	0.19	0.83	0.21	0.78	0.17
BMD: L2	0.64	0.15	0.65	0.18	0.62	0.11
BMD: L3	0.69	0.24	0.70	0.27	0.68	0.21
BMD: L4	0.81	0.20	0.83	0.21	0.79	0.19
BMD: L1-L4 total	0.76	0.19	0.78	0.21	0.73	0.17
BMD: total body	0.84	0.23	0.87	0.27	0.80	0.18

**Table 3:** Pearson correlation of VBQ in relation to DEXA and patient characteristics BMI: body mass index; BMD: body mineral density; DEXA: dual-energy X-ray absorptiometry; VBQ: vertebral bone quality; troch: trochanter; inter: intertrochanteric region

Variable	Correlation with VBQ (r)	p
Age	-0.081	0.526
Weight (kg)	0.342	0.306
Height (cm)	0.092	0.473
BMI (kg/m^2^)	-0.022	0.864
Z-score: femoral neck	-0.749	<0.0001>
Z-score: troch	-0.744	<0.0001>
Z-score: inter	-0.743	<0.0001>
Z-score: total hip	-0.742	<0.0001>
Z-score: L1	-0.776	<0.0001>
Z-score: L2	-0.736	<0.0001>
Z-score: L3	-0.736	<0.0001>
Z-score: L4	-0.735	<0.0001>
Z-score: L1-L4 total	-0.780	<0.0001>
BMD: femoral neck	-0.559	<0.0001>
BMD: troch	-0.684	<0.0001>
BMD: inter	-0.669	<0.0001>
BMD: total hip	-0.512	<0.0001>
BMD: L1	-0.611	<0.0001>
BMD: L2	-0.500	<0.0001>
BMD: L3	-0.648	<0.0001>
BMD: L4	-0.572	<0.0001>
BMD: L1-L4 total	-0.611	<0.0001>
BMD: total body	-0.516	<0.0001>

**Table 4:** Sex differences in VBQ and DEXA scores BMD: body mineral density; DEXA: dual-energy X-ray absorptiometry; MD: mean difference; SED: standard error of difference; VBQ: vertebral bone quality; troch: trochanter; inter: intertrochanteric region

Score	MD (male-female)	SED	p
VBQ score	-0.16	0.07	0.035
Z-score: femoral neck	0.29	0.43	0.500
Z-score: troch	0.90	0.41	0.032
Z-score: inter	0.95	0.42	0.027
Z-score: total hip	1.02	0.43	0.020
Z-score: L1	0.42	0.37	0.264
Z-score: L2	0.53	0.36	0.151
Z-score: L3	0.35	0.36	0.326
Z-score: L4	0.64	0.35	0.073
Z-score: L1-L4 total	0.43	0.37	0.247
BMD: femoral neck	0.05	0.07	0.495
BMD: troch	0.04	0.05	0.445
BMD: inter	0.04	0.05	0.508
BMD: total hip	0.10	0.07	0.140
BMD: L1	0.05	0.05	0.282
BMD: L2	0.03	0.04	0.385
BMD: L3	0.03	0.06	0.646
BMD: L4	0.04	0.05	0.485
BMD: L1-L4 total	0.05	0.05	0.282
BMD: total body	0.06	0.06	0.264

**Table 5:** Prediction of DEXA Z-scores from adolescent VBQ scores DEXA: dual-energy X-ray absorptiometry; R^2^: coefficient of determination; β: standardized beta coefficient; VBQ: vertebral bone quality; troch: trochanter; inter: intertrochanteric region

Predicted Z-score	R^2^	β	95% CI of β	Constant	p
Z-score: femoral neck	0.562	-4.305	-5.3/-3.3	9.064	<0.001>
Z-score: troch	0.554	-4.330	-5.3/-3.3	9.041	<0.001>
Z-score: inter	0.552	-4.426	-5.4/-3.4	9.533	<0.001>
Z-score: total hip	0.550	-4.496	-5.5/-3.5	9.728	<0.001>
Z-score: L1	0.603	-3.869	-4.7/-3.1	8.214	<0.001>
Z-score: L2	0.541	-3.621	-4.5/-2.8	7.668	<0.001>
Z-score: L3	0.542	-3.536	-4.4/-2.7	7.634	<0.001>
Z-score: L4	0.540	-3.537	-4.4/-2.7	7.400	<0.001>
Z-score: L1-L4 total	0.608	-3.929	-4.7/-3.1	8.616	<0.001>

**Table 6:** Prediction of DEXA-derived BMD scores from adolescent VBQ scores BMD: body mineral density; R^2^: coefficient of determination; β: standardized beta coefficient; DEXA: dual-energy X-ray absorptiometry; VBQ: vertebral bone quality; troch: trochanter; inter: intertrochanteric region

Predicted BMD score	R^2^	β	95% CI of β	Constant	p
BMD: femoral neck	0.312	-0.510	-0.70/-.031	1.934	<0.001>
BMD: troch	0.468	-0.498	-0.63/-0.36	1.868	<0.001>
BMD: inter	0.447	-0.485	-0.62/-0.35	1.712	<0.001>
BMD: total hip	0.263	-0.488	-0.70/-0.28	1.754	<0.001>
BMD: L1	0.373	-0.394	-0.53/-0.26	1.755	<0.001>
BMD: L2	0.250	-0.254	-0.37/-0.14	1.247	<0.001>
BMD: L3	0.420	-0.529	-0.69/-0.37	1.965	<0.001>
BMD: L4	0.328	-0.387	-0.53/-0.25	1.745	<0.001>
BMD: L1-L4 total	0.373	-0.394	-0.53/-0.26	1.705	<0.001>
BMD: total body	0.266	-0.405	-0.58/-0.23	1.813	<0.001>

## Discussion

In this study, we evaluated the association between MRI-based VBQ scores, DEXA-derived Z-scores, and BMD scores for utilization in assessing BMD in adolescent patients. We found a significant correlation between VBQ scores and DEXA-derived scores, which is the gold standard for assessing BMD in pediatric patients. We did not find any other variable, such as age or BMI, that was independently associated with the VBQ score other than DEXA-derived BMD and Z-scores. There are several other studies that show the association between VBQ scores and DEXA scores and the ability of VBQ scores to differentiate between healthy and osteoporotic or osteopenic bones [11,14,15,17,18]. However, this association is unknown in a pediatric population [16]. Ramos et al. used a VBQ score to evaluate the bone density of patients with AIS and control patients and found that those with adolescents with AIS had a higher VBQ score [16]; however, their study did not correlate a VBQ score to DEXA in their adolescent population.

To our knowledge, this is the first study to look at the correlation between VBQ scores and DEXA-derived Z-scores in a pediatric population with osteoporosis or low BMD. In this study, 63 patients were indicated for a DEXA screening for either a history of fracture or having a condition that can impact their bone quality. Ehresman et al. laid the foundation for utilizing the novel MRI-based technique to assess bone quality; this technique to obtain a VBQ score has been shown to have great interrater and intrarater reliability [19-22] and accuracy in predicting osteopenia/osteoporosis [14]. In their study using this technique to assess bone density in operative spine patients with a mean age of 66, a VBQ score of 2.74 (SD 0.60) was reported for healthy bone and 3.31 (SD 0.51) in patients with osteopenia and osteoporosis. In this study, the average VBQ score was 2.4 (SD 0.29). This VBQ score is similar to that from Ramos et al.’s study of 2.5 (SD 0.4) in their AIS patients [16]; however, it is much lower than what Ehersman et al. reported for patients with low mineral density [11]. Age may play a role here, as it is known that bone mineral accumulation varies based on sex, puberty, and genetics [23] and that generally, BMD increases from childhood to peak bone mass in early adulthood [1]. It is expected for our patient population to have a higher BMD, thus a lower VBQ, than adults over the age of 60 with osteoporosis or osteopenia.

In adolescent patients, using a DEXA-derived T-score would be inappropriate for determining BMD because peak bone mass is not yet reached. According to the International Society for Clinical Densitometry's 2013 consensus, DEXA-derived Z-scores are instead chosen to examine BMD in children, especially when assessing fracture risk [24]. Some clinicians and researchers say that BMD is considered “below the expected range for age” when Z-Scores are less than -1.0 and -2.5 [25,26]. In this study, the average DEXA Z-scores for total hip and total L1-L4 were -1.1041 and -0.8495, respectively, suggesting these patients have a BMD somewhat lower than expected. Future research should evaluate the predictive ability of the VBQ score in relation to osteopenia and osteoporosis among pediatric patients.

According to the Bone Mineral Density in Childhood Study, BMD measures show a great degree of tracking over time, suggesting that if a child has a low BMD, they may continue to have a low BMD compared to age-matched children throughout their childhood [26]. Though repeat fractures and certain conditions may put children and adolescents at risk of other injuries, Medicare will cover a repeat DEXA once every two years. MRI-derived VBQ scores can be used as a surrogate, one with no need for ionizing radiation, for tracking their BMD for these patients until they may be able to get one. This would ensure that at-risk pediatric patients are able to be followed, and we could identify and address any determinants of achieving peak bone mass and bone mineral accumulation in childhood.

There are several limitations to this study. First, the generalizability of this study may be limited due to its nature as a single-institution, retrospective study. More research will be needed to continue investigating the utility of VBQ assessment in pediatric patients. Second, the sample size in this study may be influenced by an element of selection bias due to its limited application of exclusion criteria based on patient medical history. Since we were evaluating DEXA and VBQ measurements within the same patient, we felt that patients diagnosed with a given medical condition would express uniform changes due to the given condition, as the condition’s influence on measurement would be equally detected and expressed on both assessment tools. While we felt our methodology of intra-patient analysis eliminated potential confounding caused by varying patient medical histories, nonetheless, it should be noted that the sample size in this study may be influenced by an element of selection bias.

## Conclusions

This is the first study to evaluate the correlation between VBQ scores and DEXA-derived Z-scores in a pediatric population with low BMD. Findings suggest adolescent VBQ scores are strongly associated with DEXA Z-scores and moderately associated with BMD values. The validated VBQ score used for adult patients may also provide an accurate BMD assessment for adolescent patients. VBQ has implications for predicting fracture fragility, monitoring therapeutic interventions, and ultimately improving health outcomes for adolescents. MRI-derived VBQ scores can be a more sensitive tool for assessing bone health in adolescents at risk for developing osteoporosis.

## References

[1] Rubin LA, Hawker GA, Peltekova VD, Fielding LJ, Ridout R, Cole DE (1999). Determinants of peak bone mass: clinical and genetic analyses in a young female Canadian cohort. J Bone Miner Res.

[2] Kanis JA (2002). Diagnosis of osteoporosis and assessment of fracture risk. Lancet.

[3] (2001). Osteoporosis prevention, diagnosis, and therapy. JAMA.

[4] Sözen T, Özışık L, Başaran NÇ (2017). An overview and management of osteoporosis. Eur J Rheumatol.

[5] Schuit SC, van der Klift M, Weel AE (2004). Fracture incidence and association with bone mineral density in elderly men and women: the Rotterdam Study. Bone.

[6] Zaidi Q, Danisa OA, Cheng W (2019). Measurement techniques and utility of Hounsfield unit values for assessment of bone quality prior to spinal instrumentation: a review of current literature. Spine (Phila Pa 1976).

[7] Loud KJ, Gordon CM (2006). Adolescent bone health. Arch Pediatr Adolesc Med.

[8] Keaveny TM, Clarke BL, Cosman F, Orwoll ES, Siris ES, Khosla S, Bouxsein ML (2020). Biomechanical computed tomography analysis (BCT) for clinical assessment of osteoporosis. Osteoporos Int.

[9] Jiang YW, Xu XJ, Wang R, Chen CM (2022). Radiomics analysis based on lumbar spine CT to detect osteoporosis. Eur Radiol.

[10] Engelke K (2017). Quantitative computed tomography-current status and new developments. J Clin Densitom.

[11] Ehresman J, Schilling A, Pennington Z (2019). A novel MRI-based score assessing trabecular bone quality to predict vertebral compression fractures in patients with spinal metastasis. J Neurosurg Spine.

[12] Shah LM, Hanrahan CJ (2011). MRI of spinal bone marrow: part I, techniques and normal age-related appearances. AJR Am J Roentgenol.

[13] Karampinos DC, Ruschke S, Gordijenko O (2015). Association of Mrs-based vertebral bone marrow fat fraction with bone strength in a human in vitro model. J Osteoporos.

[14] Ehresman J, Pennington Z, Schilling A (2020). Novel MRI-based score for assessment of bone density in operative spine patients. Spine J.

[15] Ehresman J, Schilling A, Yang X (2021). Vertebral bone quality score predicts fragility fractures independently of bone mineral density. Spine J.

[16] Ramos O, Razzouk J, Chung JH, Cheng WK, Danisa OA (2022). Opportunistic assessment of bone density in patients with adolescent idiopathic scoliosis using MRI-based vertebral bone quality. J Clin Neurosci.

[17] Li R, Yin Y, Ji W, Wu X, Jiang H, Chen J, Zhu Q (2022). MRI-based vertebral bone quality score effectively reflects bone quality in patients with osteoporotic vertebral compressive fractures. Eur Spine J.

[18] Kim AY, Lyons K, Sarmiento M, Lafage V, Iyer S (2023). MRI-based score for assessment of bone mineral density in operative spine patients. Spine (Phila Pa 1976).

[19] Mierke A, Ramos O, Macneille R, Chung JH, Wycliffe N, Cheng W, Danisa OA (2022). Intra- and inter-observer reliability of the novel vertebral bone quality score. Eur Spine J.

[20] Schilling AT, Ehresman J, Pennington Z (2021). Interrater and intrarater reliability of the vertebral bone quality score. World Neurosurg.

[21] Razzouk J, Ramos O, Ouro-Rodrigues E, Samayoa C, Wycliffe N, Cheng W, Danisa O (2023). Comparison of cervical, thoracic, and lumbar vertebral bone quality scores for increased utility of bone mineral density screening. Eur Spine J.

[22] Razzouk J (2023). Answer to the letter to the editor of Zhe Wang et al. concerning "Comparison of cervical, thoracic, and lumbar vertebral bone quality scores for increased utility of bone mineral density screening" by Razzouk J, Ramos O, Ouro-Rodrigues E, et al. [Eur Spine J (2022): doi:10.1007/s00586-022-07484-5]. Eur Spine J.

[23] Bachrach LK (2001). Acquisition of optimal bone mass in childhood and adolescence. Trends Endocrinol Metab.

[24] Carey JJ, Delaney MF (2010). T-scores and Z-scores. Clinic Rev Bone Miner Metab.

[25] Binkovitz LA, Henwood MJ (2007). Pediatric DXA: technique and interpretation. Pediatr Radiol.

[26] Kalkwarf HJ, Zemel BS, Gilsanz V (2007). The bone mineral density in childhood study: bone mineral content and density according to age, sex, and race. J Clin Endocrinol Metab.

